# 非小细胞肺癌免疫耐药机制及应对策略的研究进展

**DOI:** 10.3779/j.issn.1009-3419.2023.102.03

**Published:** 2023-01-20

**Authors:** Yawan JING, Hao ZENG, Ruixin CHENG, Panwen TIAN, Yalun LI

**Affiliations:** ^1^610041 成都，四川大学华西医院呼吸与危重症医学科; ^1^Department of Respiratory and Critical Care Medicine, West China Hospital; ^2^四川大学华西临床医学院; ^2^West China School of Medicine, Sichuan University; ^3^四川大学华西医院呼吸与危重症医学科/肺癌中心; ^3^Lung Cancer Center, West China Hospital, Sichuan University, Chengdu 610041, China

**Keywords:** 免疫治疗, 免疫耐药, 肺肿瘤, 应对策略, Immunotherapy, Immunoresistance, Lung neoplasms, Coping strategies

## Abstract

免疫治疗对非小细胞肺癌（non-small cell lung cancer, NSCLC）有显著的临床益处，然而，随着NSCLC免疫治疗的广泛应用，免疫耐药成为不可避免的问题。免疫治疗诱导肿瘤微环境发生广泛的细胞和分子改变，其耐药机制目前尚未完全明确，且耐药后标准化疗方案的疗效有限，亟待探索基于耐药机制的更有效的应对策略。本文拟对目前已知的免疫治疗耐药机制及应对策略进行综述，为临床医生制定更加个体化、精准化的治疗方案以及改善患者预后提供基础。

## Abstract (en)

Immunotherapy has significantly improved clinical outcomes of non-small cell lung cancer (NSCLC), however, along with the popularization of immunotherapy, immune resistance has become an unavoidable problem. Immunotherapy can induce extensive cellular and molecular alterations in the tumor microenvironment. Considering the mechanisms of immune resistance are not yet fully understood and the efficacy of standard chemotherapy regimens is limited, more effective coping strategies based on resistance mechanisms are urgently needed. In this review, we intend to summarize the known mechanisms of immune resistance and feasible strategies, so as to provide a foundation for clinicians to develop more individualized and precise regimens and finally improve patients’ prognosis.

近年来，免疫治疗（immunotherapy, IO）已成为驱动基因阴性非小细胞肺癌（non-small cell lung cancer, NSCLC）患者一线治疗的标准方案^[1,2]^。IO在晚期NSCLC中获得了长期应答，患者的5年总生存率（overall survival, OS）可达20%，而在程序性死亡配体1（programmed cell death ligand 1, PD-L1）高表达患者中高达40%^[3]^。但是，免疫耐药不可避免，本文将回顾免疫耐药的临床定义及现阶段发现的耐药机制，同时总结免疫耐药后的治疗方案。

## 1 免疫耐药定义及分类

2017年Sharma等^[4]^将晚期转移性肿瘤免疫耐药模式分为适应性耐药、原发性耐药及获得性耐药。适应性耐药指肿瘤能被免疫系统识别，但它是通过适应免疫攻击来保护自己的一种耐药机制^[4]^。原发性耐药指肿瘤对IO初始治疗无反应，患者IO无效^[4]^。获得性耐药指患者最初对IO有效，但在IO≥6个月后肿瘤复发和进展^[4,5]^。适应性免疫耐药是一种耐药机制，其参与保护正常组织不受免疫反应的有害影响，可被肿瘤利用以逃避免疫攻击，在临床上可表现为原发性耐药、获得性耐药或者二者的混合效应^[4]^。

## 2 免疫耐药机制

免疫耐药机制包括：（1）肿瘤内源性机制：包括基因及相关信号通路的改变、低肿瘤突变负荷（tumor mutational burden, TMB）与新抗原的减少、肿瘤抗原呈递减少、PD-L1的表达、表观遗传变化；（2）肿瘤外源性机制：包括免疫效应细胞、替代免疫检查点、免疫抑制细胞、肿瘤微环境（tumor microenvironment, TME）的代谢变化、免疫抑制因子、血管内皮生长因子（vascular endothelial growth factor, VEGF）；（3）宿主相关机制（图1）。

**图1:**
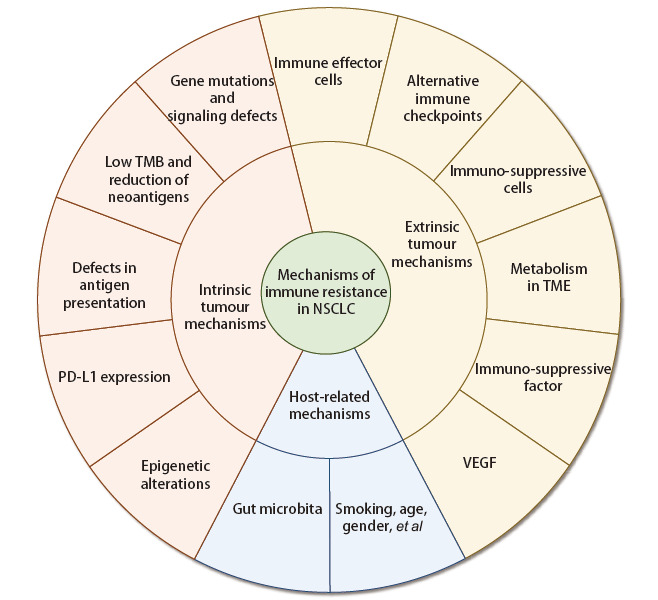
NSCLC免疫耐药机制

### 2.1 肿瘤内源性机制

#### 2.1.1 基因及相关信号通路的改变

基因突变可直接或间接通过影响相关信号通路导致NSCLC免疫耐药。10号染色体缺失的磷酸酶基因（phosphatase and tensin homolog deleted on chromosome 10, PTEN）缺失是激活磷脂酰肌醇-3-激酶（posphoinositide 3-kinase, PI3K）通路的最常见方式^[6]^，从而抑制γ-干扰素（interferon-γ, IFN-γ）、颗粒酶B的表达和T细胞的募集^[7]^。此外，PTEN缺失还导致肿瘤细胞PD-L1表达上调，最终抑制效应T细胞的功能^[8]^。间变性淋巴瘤激酶（anaplastic lymphoma kinase, ALK）融合通过激活PI3K/蛋白激酶B和丝裂原活化蛋白激酶（mitogen-activated protein kinase, MAPK）通路减少新抗原的产生，使免疫抑制细胞增殖，导致免疫耐药^[9,10]^。约10%的NSCLC具有SMARCA4的异常表达^[11]^，SMARCA4又称转录激活因子，是SWI/SNF染色质重塑复合物的ATP依赖性催化亚基，SWI/SWF复合物通过加强T细胞信号传导来调节TME以影响IO疗效^[12]^，具有SMARCA4单纯突变的NSCLC患者对程序性死亡受体1（programmed cell death 1, PD-1）/PD-L1抑制剂反应良好^[13,14]^；鼠类肉瘤病毒癌基因（Kirsten rat sarcoma viral oncogene, KRAS）突变通过增加PD-L1的表达以促进免疫逃逸^[15]^，却对激活免疫抑制细胞没有明显作用^[16]^，具有KRAS单纯突变^[17⇓-19]^的NSCLC患者对IO反应也较好；但是，对于BRG1与KRAS共突变的NSCLC患者，IO反应却更差^[20,21]^。JAK1/2突变已被证实与继发性免疫耐药相关，由于其导致IFN-γ信号丢失，并在IFN-γ暴露时阻止适应性PD-L1表达，还可导致原发性免疫耐药^[22]^。

基因突变还可通过影响TME导致NSCLC免疫耐药。表皮生长因子受体（epidermal growth factor receptor, EGFR）通过上调PD-L1表达、导致免疫抑制性TME及减少抗原呈递和新抗原的产生，从而发生原发性免疫耐药^[23,24]^。丝/苏氨酸激酶11（serine/threonine kinase 11, STK11）基因突变负调控哺乳动物雷帕霉素靶蛋白（mammalian target of rapamycin, mTOR）通路并使细胞毒性CD8^+ ^T淋巴细胞浸润减少^[25]^，导致抑制性TME。研究^[26⇓-28]^表明，STK11突变与PD-L1抑制剂度伐利尤单抗的耐药性相关，这表明STK11突变是NSCLC患者免疫耐药的潜在驱动因素。

#### 2.1.2 低TMB与新抗原的减少

TMB指肿瘤基因外显子区域每兆碱基的突变总数^[29]^。肿瘤新抗原是指在正常组织中不表达而仅存在于肿瘤组织中的抗原^[29]^。TMB增高、DNA错配修复基因缺失及高基因组微卫星不稳定性均可增加肿瘤抗原表达并提高免疫疗效^[30⇓-32]^。在NSCLC相关研究中发现，TMB与新抗原的产生、肿瘤浸润淋巴细胞（tumor infiltrating lymphocytes, TILs）的浸润及T细胞受体的多样性呈正相关^[33,34]^，通过抗肿瘤反应的免疫选择可导致新抗原丢失变体的生长和肿瘤免疫逃逸，导致免疫耐药^[35]^。这种新抗原的进化也可能发生在IO过程中，导致耐药性^[36]^。研究^[36]^发现，经帕博利珠单抗治疗且无持续临床获益的NSCLC患者的新抗原载量明显降低；该研究还发现，参与编码肿瘤抗原的新突变比例下降及新抗原丢失，从而导致获得性耐药。

#### 2.1.3 肿瘤抗原呈递减少

CD8^+ ^T细胞通过由人类白细胞抗原I（human leukocyte antigen I, HLA-I）基因编码的主要组织相容性复合体（major histocompatibility complex, MHC）I类分子呈递新抗原来识别肿瘤细胞^[37]^，B2M基因突变可导致细胞表面MHC I类分子丢失，进而导致CD8^+^ T细胞识别功能障碍，诱导NSCLC患者发生免疫耐药^[38]^。HLA-I基因杂合性的丢失导致NSCLC免疫耐药，并与IO预后较差相关^[38⇓⇓-41]^。此外，TILs中MHC II类分子表达缺失的NSCLC患者，由于无法有效激活辅助T细胞及肿瘤抗原呈递减少，也可发生免疫耐药^[42]^。

#### 2.1.4 PD-L1的表达

IO中PD-1/PD-L1抑制剂主要通过阻断PD-1/PD-L1通路以发挥作用，因此，TME中缺乏PD-L1的表达是一种直接的耐药机制^[43]^。Gao等^[44]^研究揭示了PD-L1通过去乙酰化依赖性核转位控制免疫应答相关基因表达，并调控免疫反应相关通路，包括I型和II型干扰素信号通路、核因子κB信号通路和抗原呈递通路，从而介导PD-1/PD-L1抑制剂的耐药。其中，IFN-γ是上调和维持TME中PD-L1表达的主要细胞因子，而上调的PD-L1诱导T细胞功能障碍^[45]^。此外，干扰素也是参与抗肿瘤免疫的重要细胞因子，尽管肿瘤特异性T细胞向TME不断分泌IFN-γ可能导致免疫逃逸^[46]^，但其适度表达通过增强抗原呈递、募集免疫细胞和诱导凋亡，在肿瘤细胞中产生有效的免疫反应^[47]^，干扰素通路相关基因的遗传缺陷与IO的原发性和获得性耐药均有关^[38]^。

#### 2.1.5 表观遗传变化

表观遗传机制将肿瘤相关的免疫细胞和基质细胞的环境重新编程为免疫抑制状态^[48]^，其影响免疫检查点的表达，破坏抗原呈递过程，抑制T细胞向TME的募集和T细胞的活化^[49]^。NSCLC中启动子超甲基化使含有新抗原突变的基因优先抑制，从而导致转录组新抗原耗竭^[35]^，而新抗原的耗竭介导免疫耐药。此外，外泌体中的长链非编码RNA、微小RNA和环状RNA与NSCLC等多种癌症的免疫抑制、迁移、增殖及侵袭密切相关，从而介导免疫耐药^[50⇓⇓⇓-54]^。癌细胞衍生的外泌体circUSP7通过调节NSCLC中miR-934/SHP2轴诱导CD8^+ ^T细胞功能障碍及抗PD-1治疗耐药^[55]^。N6-甲基腺苷修饰的circIGF2BP3抑制CD8^+ ^T细胞反应，通过诱导PD-L1的去泛素化，导致NSCLC肿瘤细胞免疫逃避以介导免疫耐药^[54]^。

### 2.2 肿瘤外源性机制

#### 2.2.1 免疫效应细胞

TME是指免疫细胞、基质细胞、内皮细胞和其他非细胞成分组成的高度复杂和动态的局部环境^[56,57]^。TME中免疫效应细胞浸润较少和耗竭导致免疫抑制和免疫逃逸，包括细胞毒性T淋巴细胞（cytotoxic T lymphocyte, CTL）、自然杀伤（natural killer, NK）细胞、树突状细胞（dendritic cells, DCs）等。其中CTL通过多种机制[包括IFN-γ、肿瘤坏死因子α（tumor necrosis factor α, TNF-α）、穿孔素和颗粒酶B的活性]识别和直接杀伤癌细胞，然而TME中的CTL可能存在功能失调^[58]^，并且随着时间的推移发展出免疫逃逸机制，如PD-L1表达增加，从而导致适应性免疫耐药。此外，TME中的肿瘤细胞和基质细胞都有助于T细胞的排斥^[59]^，形成物理隔绝而无法杀伤癌细胞。此外，在NSCLC的小鼠模型中发现，小鼠淋巴结中的T淋巴细胞已经出现功能失调，从而无法在肿瘤组织中发挥其免疫作用^[60]^，这可能导致免疫耐药。

#### 2.2.2 替代免疫检查点

多个替代免疫检查点的表达与T细胞耗竭相关，从而导致NSCLC免疫耐药^[61⇓-63]^。其中，淋巴细胞活化基因3（lymphocyte activation gene 3, LAG-3）、T细胞免疫球蛋白和黏蛋白结构域3蛋白（T-cell immunoglobulin, mucin domain 3 protein, TIM-3），通过抑制效应性T细胞（effective T cells, Teffs）及DCs的活化，促进调节性T细胞（regulatory T cells, Tregs）及髓源性抑制细胞（myeloid-derived suppressor cells, MDSCs）的活化及增殖，发挥免疫抑制作用^[64]^。有研究^[38]^对8例NSCLC免疫耐药患者肿瘤组织进行活检，其中5例在TILs上的LAG-3表达增加，3例出现了TIM-3表达增加。T细胞激活抑制物免疫球蛋白可变区结构域（V-domain immunoglobulin-containing suppressor of T-cell activation, VISTA）是NSCLC中具有调节免疫功能及免疫耐药性的另一种替代免疫检查点，可作为应对免疫耐药的潜在靶点^[65]^。

#### 2.2.3 免疫抑制细胞

在NSCLC临床前癌症模型中发现，Tregs^[66]^、MDSCs^[67]^和TAMs^[68]^损害抗肿瘤免疫应答。Tregs可抑制CD4^+ ^T和CD8^+ ^T细胞的活化和增殖，抑制幼稚和记忆T细胞的功能，也被可以表达自身抗原的肿瘤细胞利用以逃避免疫监视^[69]^，从而导致免疫耐药。既往已有多项研究发现，NSCLC患者肿瘤实质和TME中Tregs明显增高^[70⇓⇓-73]^，从而导致较差的免疫反应和临床疗效^[70,74⇓-76]^。

MDSCs是被招募到TME的异质骨髓细胞，具有免疫抑制功能^[67]^，可通过多种方式介导免疫逃逸和免疫耐药^[77]^。晚期NSCLC患者MDSCs异常积累与免疫耐药密切相关^[78⇓-80]^。MDSCs通过分泌IFN-γ和白细胞介素10（interleukin-10, IL-10）促进FOXP3^+^ Tregs细胞的产生，促进血管生成、肿瘤侵袭和转移，抑制Teff增殖；还通过阻断淋巴细胞归巢和调节腺苷代谢所需的酶来发挥免疫抑制作用^[78,81]^。临床前研究^[82⇓⇓-85]^表明，靶向MDSCs可以增强包括NSCLC在内的多种实体瘤的IO反应。

在NSCLC的基础研究中显示，肿瘤相关巨噬细胞（tumor-associated macrophages, TAMs）的聚集与趋化因子C-C基元配体2（chemokine C-C motif ligand 2, CCL2）的水平呈正相关^[86]^，其通过减弱抗原提呈能力及释放IL-10、转化生长因子β（transforming growth factor β, TGF-β）等免疫抑制因子，从而抑制T细胞功能^[87]^。TAMs与血管和淋巴管生成显著相关，进而导致NSCLC免疫治疗耐药^[88]^。TAMs相关受体激活也会导致NSCLC小鼠模型发生免疫耐药^[89]^。因此，靶向TAMs受体是克服NSCLC免疫耐药的潜在治疗策略，相关I期及II期临床研究正在进行中^[90]^。

#### 2.2.4 TME中的代谢变化

TME中癌细胞和免疫细胞的代谢及它们之间的相互作用均会导致肿瘤的进展和转移^[91⇓⇓-94]^。精氨酸酶主要由MDSCs在局部刺激（如免疫抑制细胞因子、缺氧、酸中毒）下产生，可阻碍T细胞功能，并抑制T细胞增殖和分化^[95]^。有研究^[96,97]^在NSCLC耐药模型和人群的肿瘤细胞及肿瘤基质中观察到精氨酸酶显著升高，并在肺癌小鼠模型中验证后发现精氨酸酶是可调节的免疫靶点。但是目前的证据大多来自于临床前研究，尚需进一步探索TME中的代谢失衡与免疫耐药机制的相关性。

#### 2.2.5 免疫抑制因子

肿瘤细胞、免疫细胞、基质细胞通过产生免疫抑制因子，如TGF-β、腺苷、吲哚胺2,3-双加氧化酶（indoleamine 2,3-dioxygenase, IDO），形成免疫抑制性TME，从而损伤免疫应答。

TME中的癌细胞、肿瘤相关成纤维细胞（cancer associated fibroblasts, CAFs）和其他细胞释放的TGF-β通过塑造肿瘤结构、抑制免疫细胞的抗肿瘤活性、促进癌细胞的侵袭和扩散、诱导癌细胞的干细胞特性，促进免疫抑制微环境及肿瘤进展，导致免疫耐药^[98⇓⇓-101]^。目前，在NSCLC等多种癌症类型中TGF-β联合抗PD-1/PD-L1治疗的多项临床试验正在进行中^[101]^。

腺苷可抑制Teff和NK细胞、增加Tregs和MDSCs的浸润、促进CAFs的增殖，从而导致抑制性TME^[92]^。因此，阻断腺苷通路是应对免疫耐药可行的方法。DPCPX（一种腺苷受体拮抗剂）已显示出与PD-1抗体在NSCLC患者中的协同作用^[102]^。此外，通过阻断CD39和CD73的活性来消耗细胞外腺苷，也可能阻断腺苷信号通路，相关I期/II期临床研究正在进行中（NCT03381274, NCT03454451）。

IDO过表达导致犬尿氨酸的积累和色氨酸的消耗，从而促进Tregs和MDSCs生成，并且抑制Teff的增殖和激活^[103]^。研究^[104]^表明，在临床前PD-1/PD-L1抑制剂治疗肺腺癌耐药模型中，TILs中IDO1高表达与抗PD-1/PD-L1治疗耐药有关，IDO抑制剂的应用可下调IDO的表达并增加CD8^+^ T细胞的浸润，从而重新激活T细胞的抗肿瘤反应。

#### 2.2.6 VEGF

VEGF是诱导肿瘤微血管系统生成的主要介质，可以通过干扰DCs成熟而抑制抗原呈递，进而抑制T细胞活化，诱导CD8^+ ^T细胞耗竭，激活Tregs和TAMs，从而导致免疫抑制TME，与NSCLC的进展、复发、转移和免疫耐药有关^[105⇓⇓-108]^。目前，VEGF或VEGF受体（VEGF receptor, VEGFR）的中和抗体和受体酪氨酸激酶抑制剂（tyrosine kinase inhibitors, TKIs）作为克服免疫耐药的策略，不管单独或者联合IO，已在NSCLC患者中成熟应用^[107,109⇓-111]^。

### 2.3 宿主相关机制

肠道微生态在局部和全身宿主免疫应答中起重要作用^[112]^。NSCLC对免疫治疗的反应与肠道微生物群密切相关^[113⇓-115]^。Routy等^[114]^研究了嗜黏蛋白阿克曼菌在NSCLC中对PD-1/PD-L1抑制剂的反应和耐药性的预测作用，发现其相对丰度高可能提示对IO耐药。Oster等^[116]^发现幽门螺杆菌血清阳性与抗PD-1治疗的NSCLC患者生存率及无进展生存率明显降低相关。研究^[117]^发现，人参多糖（Ginseng polysaccharides, GPs）联合αPD-1单抗可将NSCLC小鼠模型肠道微生物群从无应答型重塑为应答型，可能是NSCLC患者克服PD-1抑制剂耐药的潜在治疗策略。基于以上研究基础，肠道菌群移植、活性益生菌、膳食纤维及益生元等多种调控肠道菌群的策略可能为克服NSCLC免疫耐药带来新的希望，部分针对NSCLC患者的I期/II期临床研究正在进行中（NCT03637803）。

此外，吸烟、性别及体型肥胖也与NSCLC患者IO治疗疗效相关。研究^[118⇓-120]^显示，吸烟患者比不吸烟患者对IO应答更好，这可能与香烟中的苯并芘能够介导肺上皮细胞PD-L1表达增加以及吸烟者基因突变频率比不吸烟者高10倍以上有关。一项针对接受ICI治疗的肿瘤患者的meta分析^[121]^显示男性患者接受IO效果更佳。体型肥胖的患者接受PD-L1抑制剂治疗，其OS显著提高，死亡风险降低^[122]^。

## 3 免疫耐药后的应对策略

### 3.1 针对免疫治疗后特殊反应类型的应对策略

当免疫治疗出现“假性进展、分离反应、延迟反应”的非典型反应特征后^[123⇓⇓-126]^，继续IO仍然能够提高临床获益^[127⇓-129]^，但必须评估符合继续IO的患者。OAK研究^[128]^提示，进展后继续使用阿替利珠单抗治疗组，相比于换用其他治疗或无治疗组，获益显著，18个月OS率为37%，中位OS为12.7个月。基于IO的非典型反应特征，III期临床研究中针对特定人群，允许IO进展后继续治疗^[130,131]^。因此，免疫治疗后在特殊反应类型中再挑战IO是一种可行的策略^[132]^。

### 3.2 针对免疫治疗后寡进展的应对策略

临床中IO获得性耐药以寡进展为主，寡进展的患者有望从局部消融治疗（local ablation treatment, LAT）中获益^[133]^，是克服获得性耐药的潜在治疗策略。目前已有关于LAT在IO后NSCLC患者中应用的研究，包括放疗、热消融^[134]^，但临床证据主要来自于回顾性研究^[135,136]^。为了使局部治疗的获益最大化，优势人群的选择、局部治疗的模式选择（放疗与其他消融技术）至关重要。针对接受IO后寡进展的晚期NSCLC患者，多项临床研究正在探索中^[137]^。

### 3.3 针对免疫治疗后广泛进展的应对策略

针对NSCLC免疫耐药广泛进展的应对策略正在迅速发展，多种治疗手段协同作用以克服免疫耐药^[138⇓-140]^，包括IO联合免疫共刺激信号（CD40、OX40、GITR等）、IO联合TME调节治疗（VEGFR-TKIs、TGF-β/PD-L1双特异性抗体、NKTR-214等）、IO联合T细胞相关治疗[放疗、化疗、抗体药物偶联物（antibody-drug conjugate, ADC）、癌症疫苗等]、IO联合共抑制信号[LAG-3、TIM-3、VISTA、细胞毒性T淋巴细胞相关蛋白4（cytotoxic T-lymphocyte-associated protein 4, CTLA-4）等]、IO联合基于微生物组的治疗，见表1。探索新的检查点抑制剂和治疗组合的临床试验也在不断进行中，见表2。晚期NSCLC免疫治疗耐药后，根据不同进展类型的治疗策略如图2。

**表1:** 基于耐药机制的NSCLC免疫耐药应对策略

Strategies	Targets/method	R&D status
IO+costimulatory signals	CD40	Phase II
	OX40	Phase I/II
	GITR	Phase I/II
	CD137/4-1BB	Phase I
	ICOS	Phase II
IO+regulating TME	VEGFR-TKIs	Phase III
	VEGFR-TKIs+chemotherapy	Phase III
	TGF-β/PD-L1 bi-specific antibodies	Phase III
	IL-1β inhibitory	Phase III failure
	IL-2 (NKTR-214)	Phase I/II
	IL-15 stimulatory	Phase III
	CXCR2	Phase II
	CD73	Phase II
	CDK4/6	Phase I
IO+T cell associated therapies	Radiotherapy	FDA-approved
	Chemotherapy	FDA-approved
	ADC	Phase III
	Cancer vaccines	Phase II
	Adoptive cell therapy	Phase II
	STING stimulatory	Phase I
	TLR stimulatory	Phase I
IO+coinhibitory signals	CTLA-4	FDA-approved
	TIGIT	Phase III
	LAG-3	Phase II
	TIM-3	Phase I/II
	NKG2A	Phase II
	VISTA	Phase I
	Siglec-15	Phase I/II
IO+microbiome-based therapy	Microbiome	Phase I/II

NCT: National Clinical Trial; GITR: glucocorticoid-induced tumor necrosis factor receptor; ICOS: inducible T cell co-stimulator; VEGFR: VEGR receptor; TKIs: tyrosine kinase inhibitors; TGF-β: transforming growth factor β; IL: interleukin; CXCR2: C-X-C motif chemokine receptor 2; CDK4/6: cyclin-dependent kinase 4/6; FDA: Food and Drug Administration; ADC: antibody-drug conjugate; STING: stimulator of interferon gene; TLR: Toll-like receptor; CTLA-4: cytotoxic T lymphocyte-associated protein 4; TIGIT: T-cell immunoreceptor tyrosine-based inhibition motif domain; NKG2A: natural killer group 2 member A; Siglec-15: sialic acid-binding Ig-like lectin 15; VISTA: V-domain Immunoglobulin-containing suppressor of T-cells activation.

**表2:** 应对NSCLC免疫耐药策略的部分临床研究汇总

New strategy	Compound	Regimens	NCT number; Phase
IO+chemotherapy	Pembrolizumab	Pembrolizumab+chemotherapy vs chemotherapy	NCT03793179; Phase III
IO+anti-angiogenesis	Sitravatinib	Tislelizumab+Sitravatinib vs Docetaxel	NCT04921358; Phase III
	Sitravatinib	Nivolumab+Sitravatinib vs Docetaxel	NCT03906071; Phase III
	Cabozantinib	Atezolizumab+Cabozantinib vs Docetaxel	NCT04471428; Phase III
	Lenvatinib	Pembrolizumab+Lenvatinib vs Docetaxel	NCT03976375; Phase III
	Famitinib	Camrelizumab+Famitinib vs Docetaxel	NCT05106335; Phase III
IO+IL-15	ALT-803	Pembrolizumab+ALT-803 vs standard therapy	NCT05096663; Phase II/III
IO+PARPi	Oaparib	Durvalumab+Olaparib	NCT03334617; Phase II
IO+CD73	CPI-006	CPI-006 vs CPI-006+Ciforadenant/Pembrolizumab	NCT03454451; Phase I
	PT199	PT199 vs PT199+anti-PD-1 monoclonal antibody	NCT05731270; Phase I
ADC	DS-1062A	DS-1062a vs Docetaxel	NCT04656652; Phase III
	SG	SG vs Docetaxel	NCT05089734; Phase III
Vaccines and ACT	OSE2101	OSE2101 vs Docetaxel/Pemetrexed	NCT02654587; Phase III
	TG4010 (MVA-MUC1-IL2 vaccine)	TG4010+Nivolumab	NCT02823990; Phase II
	WT1-TCR CD8^+^ T cells	NA	NCT02408016; Phase I/II
	MUC1 CAR-T cells	CAR-T with or without PD-1 knockout T cells	NCT03525782; Phase I/II
IO+microbiome-based therapy	MRx0518	MRx0518+Pembrolizumab	NCT03637803; Phase I/II
	SYNB1891	SYNB1891+Atezolizumab	NCT04167137; Phase I
	GEN-001	GEN-001+Avelumab	NCT04601402; Phase I

SG: Sacituzumab Govitecan-hziy; PARPi: poly ADP-ribose polymerase inhibitor; ACT: adoptive cell therapy; NA: not available; CAR-T: chimeric antigen receptor T cell; PD-1: programmed cell death 1.

**图2:**
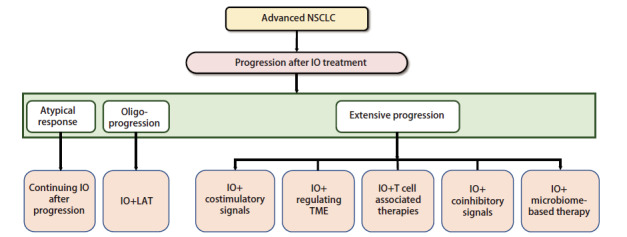
晚期NSCLC免疫耐药后的治疗选择流程图

## 4 结语

目前NSCLC免疫耐药机制的研究仍在不断探索中，再活检率低和生物样本量有限是免疫耐药机制研究中的难点。未来需要继续深入探索免疫耐药机制及相关通路，评估耐药机制的分层及主导地位以及与TME的相互关系，明确TME向免疫促进方向转化的关键靶点，为患者选择和逆转免疫耐药的应对策略提供新思路。
